# Early cessation of breastfeeding amongst women in South Africa: an area needing urgent attention to improve child health

**DOI:** 10.1186/1471-2431-12-105

**Published:** 2012-07-24

**Authors:** Tanya Doherty, David Sanders, Debra Jackson, Sonja Swanevelder, Carl Lombard, Wanga Zembe, Mickey Chopra, Ameena Goga, Mark Colvin, Lars T Fadnes, Ingunn MS Engebretsen, Eva-Charlotte Ekström, Thorkild Tylleskär

**Affiliations:** 1 Medical Research Council, Francie van Zyl Drive, Parow, Cape Town, South Africa; 2 University of the Western Cape, Modderdam Road, Bellville, Cape Town, South Africa; 3 UNICEF, UNICEF House, 3 United Nations Plaza, New York, NY, 10017, USA; 4 Centre for International Health, University of Bergen, Årstadveien 21 5th floor, Bergen, N-5009, Norway; 5 Department of Women’s and Children’s Health, Uppsala University, Drottninggatan 4, 4th level, Uppsala, Sweden; 6 Maromi Health Research, 27 Cohen Avenue, Glenwood, Durban, 4083, South Africa; 7 Department of Child and Adolescent Psychiatry, Haukeland University Hospital, Bergen, Norway

## Abstract

**Background:**

Breastfeeding is a critical component of interventions to reduce child mortality. Exclusive breastfeeding practice is extremely low in South Africa and there has been no improvement in this over the past ten years largely due to fears of HIV transmission. Early cessation of breastfeeding has been found to have negative effects on child morbidity and survival in several studies in Africa. This paper reports on determinants of early breastfeeding cessation among women in South Africa.

**Methods:**

This is a sub group analysis of a community-based cluster-randomized trial (PROMISE EBF) promoting exclusive breastfeeding in three South African sites (Paarl in the Western Cape Province, and Umlazi and Rietvlei in KwaZulu-Natal) between 2006 and 2008 (ClinicalTrials.gov no: NCT00397150). Infant feeding recall of 22 food and fluid items was collected at 3, 6, 12 and 24 weeks postpartum. Women’s experiences of breast health problems were also collected at the same time points. 999 women who ever breastfed were included in the analysis. Univariable and multivariable logistic regression analysis adjusting for site, arm and cluster, was performed to determine predictors of stopping breastfeeding by 12 weeks postpartum.

**Results:**

By 12 weeks postpartum, 20% of HIV-negative women and 40% of HIV-positive women had stopped all breastfeeding. About a third of women introduced other fluids, most commonly formula milk, within the first 3 days after birth. Antenatal intention not to breastfeed and being undecided about how to feed were most strongly associated with stopping breastfeeding by 12 weeks (Adjusted odds ratio, AOR 5.6, 95% CI 3.4 – 9.5 and AOR 4.1, 95% CI 1.6 – 10.8, respectively). Also important was self-reported breast health problems associated with a 3-fold risk of stopping breastfeeding (AOR 3.1, 95%CI 1.7 – 5.7) and the mother having her own income doubled the risk of stopping breastfeeding (AOR 1.9, 95% CI 1.3 – 2.8).

**Conclusion:**

Early cessation of breastfeeding is common amongst both HIV-negative and positive women in South Africa. There is an urgent need to improve antenatal breastfeeding counselling taking into account the challenges faced by working women as well as early postnatal lactation support to prevent breast health problems.

## Background

South Africa is one of five countries in Southern Africa that is considered to have made no progress between 1990 and 2008 in reducing mortality amongst children younger than five years (Millennium Development Goal 4) [1]. In this period, the under-5-mortality rate rose from 56 to 67 per 1000 live births, with 57 percent of all child deaths attributable to HIV/AIDS, followed by pneumonia, diarrhoea and undernutrition [2].

Even before the emergence of the HIV epidemic in South Africa, breastfeeding practices were sub optimal. Although initiation of breastfeeding is high, Demographic and Health Surveys have reported a decline in initiation from 87% in 1998 [3] to 82% in 2003 [4]. Exclusive breastfeeding in South Africa is extremely low and has not improved over the past thirteen years; 7% under six months in 1998 [3] and 8% under 6 months in 2003 [4].

In 2001, with the introduction of the Prevention of Mother to Child HIV Transmission (PMTCT) programme guidelines were released by the National Department of Health regarding infant feeding advice to be given to HIV-positive women. Given the existing knowledge at the time, those guidelines recommended abrupt cessation of breastfeeding at four months for HIV- positive women who chose to breastfeed [5]. Furthermore provision was made for free formula milk to be provided from public health facilities for HIV-positive women choosing not to breastfeed. This policy did not seem unusual in South Africa, where formula milk has been dispensed for many years as part of the ‘Protein Energy Malnutrition scheme’ to rehabilitate malnourished children [6], and in which there has been no enforcement of, or legislation based on, the International Code of Marketing Breast Milk Substitutes. Furthermore, from 2001 to present there has been no investment in advocacy or media to promote breastfeeding, partly due to fears of HIV transmission through breast milk [7]. When the PMTCT programme was first implemented there was very little evidence [8] regarding the risk of HIV transmission with different breastfeeding practices; hence any type of breastfeeding (exclusive or mixed) was feared to carry a large risk of HIV transmission and research across Africa has found that health workers commonly overestimate this risk when counselling mothers [9,10].

The World Health Organisation (WHO) recommends exclusive breastfeeding during the first six months of life for optimal growth, development and health. Breastfeeding should continue up to two years or more and nutritionally adequate, safe, and appropriately-fed complementary foods should be introduced at the age of six months to meet the evolving needs of the growing infant [11]. The WHO recommendation at the time of this study regarding infant feeding for HIV positive women was exclusive breastfeeding for the first six months of life unless replacement feeding is acceptable, feasible, affordable, sustainable and safe [12].

Exclusive breastfeeding could make a large contribution to child survival, being the single most effective intervention for reducing under five mortality in low income settings [13]. Analyses of risks associated with partial or no breastfeeding have suggested that high coverage of promotion of exclusive breastfeeding could lead to a 11.6% reduction in the number of infant deaths and avert 21.9 million disability adjusted life years (8.6%) [14]. Early cessation or not breastfeeding is therefore associated with disadvantages in terms of survival and morbidity amongst both HIV exposed and unexposed infants [15-19].

Poor breastfeeding technique especially latching and positioning are known to predispose women to breast health problems including mastitis, cracked nipples and engorgement [20]. These conditions are known to interfere with the success and duration of optimal breast-feeding practice in the first 6 months after birth [21] and in the case of sub clinical mastitis, increasing the risk of postnatal HIV transmission [22-24].

This paper reports on factors associated with cessation of breastfeeding by 12 weeks postpartum amongst HIV-positive and negative women in South Africa using infant feeding information from a large multi country community randomised trial known as PROMISE EBF. The trial aimed to increase the practice of exclusive breastfeeding through supportive counselling to mothers during home visits by community peer supporters. The intervention had effects of public health significance in terms of substantial behavioural changes in two of the countries where EBF prevalence at 12 weeks in the intervention and control groups were respectively 79.1% and 34.6% in Burkina Faso and 81.6% and 43.9% in Uganda [25]. In South Africa, a similar relative increase was noted (10.5% and 6.2%) but the public health benefit is unlikely to be significant since the absolute change was small. The aim of this paper is to describe determinants of cessation of breastfeeding by 12 weeks which need to be addressed in order to improve the success and public health impact of breastfeeding promotion programmes in South Africa.

## Methods

This study used infant feeding data from a community-based cluster-randomized trial promoting exclusive breastfeeding by peer-counsellors in three South African sites between 2006 and 2008 (ClinicalTrials.gov no: NCT00397150). A total of 34 clusters from three areas in South Africa were chosen: Paarl in Western Cape Province (peri-urban), and Umlazi (urban) and Rietvlei (rural) in KwaZulu-Natal Province. Infant mortality rate (IMR) and antenatal HIV prevalence at the time of the study were 40/1000 and 10% in the Paarl site, 60/1000 and 42% in the Umlazi site and 99/1000 and 34% in Rietvlei site [4,26]. A detailed description of the methodology for the trial is described elsewhere [25]. In short, mothers in the intervention clusters received one antenatal and four postnatal visits at weeks 1, 4, 7 and 10 postpartum by peer counsellors promoting and supporting exclusive infant feeding practices (breast or formula). For breastfeeding women, peer counsellors were trained to support and encourage exclusive breastfeeding for the first six months. In the control clusters mothers received the same number of visits from peer counsellors who provided information to mothers about accessing social welfare grants. This information was not believed to have any impact on infant feeding practices.

Recruitment was a two-stage procedure. In the last trimester of pregnancy, a total of 1276 women were recruited from the three South African sites. At the 3-week assessment the mother-infant pairs were assessed and a total of 139 were excluded due to relocation or being lost-to-follow-up (95), stillbirth (9), twin delivery (11) or death of the infant (23) or mother (1) before 3 weeks after birth. Thus, 1137 mother-infant pairs were enrolled into the study. The mother-infant pairs were scheduled to be interviewed at recruitment late in pregnancy and at 3, 6, 12 and 24 weeks after birth. Interview visits were regarded as timely if they were done in the following time ranges: 3 weeks (1.5–4.5); 6 weeks (4.5–9); 12 weeks (9–18).

The recruitment interview focused mainly on socio-economic wealth and socio-demographic characteristics. The follow-up interviews addressed infant feeding using 24 hour, 7 day and since birth recall of 22 specified fluid and food items, mother’s experiences of breast health problems including cracked nipples, engorgement, infection and abscess, infant morbidity and anthropometric measurements.

### Data management

Data was collected through interviews by trained data collectors who were kept uninformed about the allocation assignment and kept separate from the intervention team. Data was subsequently double entered using EpiData (version 3.1) and analysis was done with Stata (version 10) and SAS (version 9.2).

### Analysis

Of the 1137 women enrolled in the study only women who ever breastfed were included in this analysis (n = 999). Complete cessation of breastfeeding by 3 weeks was defined as no breastfeeding in the 24 hours and 7 days prior to the 3 week interview and no breastfeeding reported (24 hour and 7 day recall) at the 6, 12 and 24 week interview. Complete cessation of breastfeeding by 6 weeks was defined as no breastfeeding in the 24 hours and 7 days prior to the 6 week interview and no breastfeeding reported (24 hour and 7 day recall) at the 12 and 24 week interview. Complete cessation of breastfeeding by 12 weeks was defined as no breastfeeding in the 24 hours and 7 days prior to the 12 week interview and no breastfeeding reported (24 hour and 7 day recall) at the final 24 week interview. Pooled estimates for the prevalence of stopping breastfeeding were calculated over the three sites. These were unweighted estimates since the sites and clusters included in the trial were purposely selected. Breast health problems were recorded at 3, 6, 12 and 24 weeks postpartum. The data collected at the 6 week interview was used for this analysis and included problems occurring between birth and 6 weeks.

A composite measure of household socio economic status was constructed with the use of multiple correspondence analysis based on ownership of assets including mobile phone and television, and house characteristics including water source, roof material and toilet type. This method is analogous to principal component analysis, and better suited for categorical data [27]. The children’s families were grouped into quintiles on the basis of socioeconomic rank.

Univariable and multivariable logistic regression analysis was performed. All variables in Table 1 (baseline characteristics) as well as explanatory variables that were either clinically (e.g. breast health problems) or epidemiologically (e.g. mothers HIV status) important were evaluated as potential determinants of early breastfeeding cessation. Variables were retained in the models if they were either at least of marginal significance or played a confounding role [28]. The variable breast problems included a category of women missing at the six weeks interview (unable to be located for an interview) to assess the effect of loss to follow up since this was the only variable in the model that was not collected at baseline (recruitment interview in late pregnancy). Model 1 includes the most proximal independent predictors (infant feeding intention, breast problems and HIV status) and model two includes the factors in model one as well as socio economic related factors (mother having own source of income and site). The models were adjusted for cluster and arm to account for the community randomised trial design. The Hosmer-Lemeshow test indicated a good model fit (0.23).

**Table 1:** Baseline characteristics of mothers who initiated breastfeeding by site

	**Paarl (n = 330)**	**Rietvlei (n = 291)**	**Umlazi (n = 378)**
Mothers age Median years	24 (20–29)	22 (19–28)	22 (19–28)
Mothers education Median years	10 (9–11)	9 (8–11)	11 (10–12)
Marital status			
Married	61 (18.5)	167 (57.9)	14 (3.7)
Cohabiting	13 (3.9)	4 (1.4)	68 (17.9)
Single/ divorced/ widowed/ separated	256 (77.6)	117 (40.6)	296 (78.3)
Missing	0	3 (1.0)	0
Parity			
Primipara	157 (47.6)	123 (42.3)	210 (55.6)
Multipara	173 (52.4)	168 (57.7)	168 (44.4)
Attendance at antenatal clinic			
Yes	324 (98.2)	278 (97.5)	377 (99.7)
SES quintile			
1 (poorest)	0	175 (60.1)	10 (2.6)
2	35 (10.7)	82 (28.2)	65 (17.2)
3	82 (25.0)	25 (8.6)	90 (23.8)
4	118 (35.9)	3 (1.0)	87 (23.0)
5 (least poor)	93 (28.3)	0	107 (28.3)
Missing	2 (0.6)	6 (2.1)	19 (5.0)
Electricity in the house			
Yes	311 (94.2)	174 (59.8)	350 (92.6)
Water source			
Surface water and other	0	216 (74.2)	1 (0.2)
Borehole/public tap	8 (2.4)	45 (15.5)	72 (19.0)
Piped into yard or house	320 (97.5)	29 (10.0)	304 (80.4)
Missing	2 (0.6)	1 (0.3)	1 (0.2)
Type of toilet			
None/open	0	99 (34.0)	47 (12.4)
Pit/ Ventilated Improved Pit	167 (50.6)	155 (53.3)	167 (44.2)
Flush	159 (48.2)	0	156 (41.3)
Missing / other	4 (1.2)	37 (12.7)	8 (2.1)

Data are number (%) or median (IQR).

### Ethics

Ethical approval was granted by the Ethics Committee of the Medical Research Council South Africa. Signed or thumb-printed informed consent was obtained from each mother prior to study participation. This study is registered with ClinicalTrials.gov, number NCT00397150.

## Results

### Baseline characteristics of breastfeeding women

Table 1 shows baseline characteristics of women by study site. The median maternal age was 22 years in Rietvlei and Umlazi and 24 years in Paarl. Completed years of education ranged from a median of 9 in Rietvlei to 11 in Umlazi. The majority of women in Rietvlei were married whilst most women in Paarl and Umlazi were single. Over 95% of women in all three sites had attended antenatal care. Large differences are noted (as expected due to purposive selection) in socio economic status and access to basic infrastructure (electricity, piped water and flush toilet) between the three sites with Rietvlei being notably poorer compared to Paarl and Umlazi.

### Infant feeding practices between birth and 12 weeks amongst breastfeeding women

**Figure 1:**
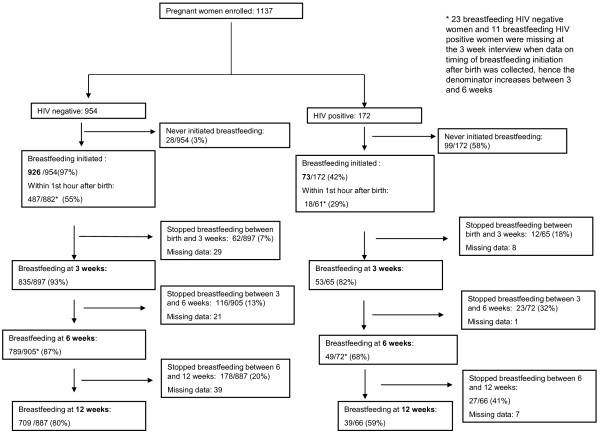
Flow chart of breastfeeding initiation and cessation at three, six and twelve weeks postpartum.

Figure 1 shows a flow chart of breastfeeding initiation and cessation amongst HIV-negative and positive women at birth, 3, 6 and 12 weeks postpartum. Almost all (97%) HIV-negative women and less than half (42%) of the HIV-positive women ever breastfed while the remainder never breastfed their infants. Seventy two (8%) HIV-negative and 28 (38%) HIV- positive women who breastfed, had intended antenatally not to breastfeed.

Of the women who breastfed their infants, just over half of the HIV-negative women and almost a third of HIV-positive women initiated breastfeeding within one hour of birth. Within the first three days after birth, 276 (31%) HIV-negative women and 22 (37%) HIV-positive women had given their infants something other than breast milk to drink, most commonly sugar water (32%), formula milk (25%) and water (21%). By three weeks postpartum, 62 (7%) and 12 (18%) HIV-negative and positive women respectively had stopped breastfeeding and by six weeks postpartum this had increased to 116 (13%) and 23 (32%) respectively. By 12 weeks 178 (20%) HIV-negative women and 27 (41%) HIV-positive women who initiated breastfeeding had stopped all breastfeeding (Figure 1).

### Predictors of stopping breastfeeding by 12 weeks

Table 2 shows the univariable and multivariable analyses of breastfeeding cessation by 12weeks. The most important predictors of stopping breastfeeding were an intention to not breastfeed antenatally (AOR 5.6, 95% CI 3.4 – 9.4) or being undecided with regard to feeding intention (AOR 4.1, 95% CI 1.5 – 10.8), breast health problems (AOR 3.1, 95% CI 1.7 – 5.7), and mother having her own source of income (AOR 1.9, 95% CI 1.3 – 2.8). Being HIV positive, although predictive on univariable analysis, was no longer significant after adjusting for antenatal feeding intention and breast problems. Adjusting for study site and arm did not alter the magnitude of the main effects (final model 2), hence the peer counselling intervention had no effect on early breastfeeding cessation.

**Table 2:** Logistic regression models of determinants of breastfeeding cessation by 12 weeks with unadjusted and adjusted odds ratios (OR) with 95% confidence intervals

	**n(%) stopping breastfeeding by 12 weeks**	**Unadjusted crude OR (95% CI)**	**Model 1 Adjusted OR (95% CI)**	**Model 2 Final adjusted OR (95% CI)**
Breast problem or infection between birth and 6 weeks				
No	130/715 (18.2)	1	1	1
Yes	38/90 (42.2)	3.2 (2.0 -5.3)	3.0 (1.7 -5.4)	3.1 (1.7 -5.7)
Missing	37/148 (25.0)	1.5 (1.0 2.2)	1.4 (0.9 – 2.1)	1.4 (0.9 – 2.3)
Antenatal feeding intention				
EBF	63/390 (15.9)	1	1	1
PBF/MF	71/404 (17.6)	1.1 (0.7 – 1.6)	1.2 (0.8 – 1.6)	1.3 (0.9 – 1.8)
Not BF	51/94 (54.3)	6.2 (3.8 – 10.2)	5.6 (3.5 – 8.7)	5.6 (3.4 – 9.4)
Don’t know	9/21 (42.9)	3.9 (1.6 – 9.8)	3.9 (1.5 – 9.7)	4.1 (1.5 – 10.8)
Mothers HIV status				
Negative	178/888 (20.1)	1	1	1
Positive	27/66 (40.9)	2.7 (1.6 – 4.6)	1.7 (0.9 – 3.0)	1.7 (1.0 – 2.8)
Mother earns money for herself				
No	115/620 (18.6)	1		1
Yes	84/295 (28.5)	1.7 (1.2 - 2.4)		1.9 (1.3 – 2.8)
Mothers age				
≤ 24	127/572 (22.2)	1		
25–29	42/191 (21.3)	0.9 (0.7 – 1.5)		
30–34	23/103 (22.3)	1.0 (0.6 – 1.7)		
≥ 35	13/85 (15.3)	0.6 (0.3 – 1.2)		
Mothers education				
Some primary school	18/97 (18.6)	1		
Some high school	112/571 (19.6)	1.1 (0.6 – 1.9)		
Completed high school	61/240 (25.4)	1.5 (0.8 – 2.7)		
Tertiary education	13/37 (35.1)	2.3 (1.0 – 5.5)		
Marital status				
Married	40/231 (17.3)	1		
Cohabiting	21/80 (26.3)	1.7 (0.9 – 3.1)		
Single/ divorced/ widowed/ separated	144/640 (22.5)	1.4 (0.9 – 2.0)		
SES quintile				
1 (poorest)	26/170 (15.3)	1		
2	27/170 (15.9)	1.0 (0.58 – 1.8)		
3	41/195 (21.0)	1.4 (0.8 – 2.5)		
4	46/204 (22.6)	1.6 (0.9 – 2.7)		
5 (least poor)	50/191 (26.2)	1.9 (1.1 – 3.3)		
Electricity in the house				
No	24/149 (16.1)	1		
Yes	181/805 (22.5)	1.5 (0.9 – 2.4)		
Study site				
Paarl	59/326 (18.1)	1		1
Rietvlei	51/269 (19.3)	1.1 (0.7 – 1.6)		1.0 (0.6 – 1.8)
Umlazi	94/357 (26.3)	1.6 ( 1.1 – 2.3)		1.1 (0.6 – 1.8)
Study arm				
Control	105/463 (22.7)	1		1
Intervention	100/490 (20.4)	0.8 (0.6 – 1.3)		1.0 (0.7 – 1.4)

All factors are cluster adjusted.

### Breast health problems

Overall 92 (11%) breastfeeding women experienced a breast health problem between birth and 6 weeks postpartum. Of these women 73 specified the type of problem they experienced and the most common problems reported were cracked nipples (35/73; 48%) followed by engorgement (16/73; 22%) and infection (15/73; 20%). There were differences in the prevalence of breast health problems by site, with 56 women in Umlazi (17%) experiencing a breast health problem compared to 19 in Rietvlei (9%) and 17 in Paarl (6%) (p = 0.00), possibly due to the higher HIV prevalence in the Umlazi site. Breast health problems were more common amongst HIV-positive women (13/63; 21%) compared to negative women (79/778; 10%) (p = 0.01) and amongst women who were mixed feeding (82/688; 12%) compared with predominantly breastfeeding (breastmilk with addition of water and other non nutritive liquids) (9/155; 6%) (p = 0.02).

## Discussion

To our knowledge, this study is the first to describe factors associated with early cessation of breastfeeding amongst a large, mostly HIV-negative sample of women in South Africa. The study has revealed sub-optimal early feeding practices, starting with low initiation of breastfeeding within one hour of birth, early introduction of other fluids and a steady increase in women stopping all breastfeeding between birth and twelve weeks postpartum. An antenatal intention not to breastfeed or being undecided about how to feed were the strongest predictors of breastfeeding cessation by 12 weeks. Experiencing a breast health problem and the mother having her own source of income also increased the likelihood of stopping breastfeeding by three and two fold respectively.

Over two thirds of the breastfeeding HIV-positive women had intended antenatally not to breastfeed and an intention not to breastfeed was found to be the strongest predictor of early breastfeeding cessation. The peer counsellors in the PROMISE intervention did not counsel women on infant feeding choices since that was the role of health care workers in the antenatal clinics. The peer counsellors supported the choice that the women had made based on the antenatal counselling received. Qualitative research undertaken during the PROMISE study [29] found that despite the availability of free formula milk for HIV-positive women, stigma associated with the collection of the free milk discourages women from carrying out their intention to not breastfeed.

Poor quality of antenatal infant feeding counselling for HIV-positive women has been well described in South Africa [7,30]; the concerning finding from this study, however, is that even in the presence of postnatal home visits from peer breastfeeding counsellors, early introduction of formula milk and a steady increase in breastfeeding cessation amongst HIV negative women could not be prevented. This scenario is unique, as in the rest of Africa, formula milk is uncommon in the first six months. Our study found that women who had their own source of income were twice as likely to stop breastfeeding by 12 weeks. This income may be related to employment with little or no maternity leave provision. It would also enable the purchase of formula milk and a means to replace breastmilk. A cross sectional survey of feeding practices amongst HIV-positive women in Uganda also found that women who were socio-economically better off were more likely to stop breastfeeding earlier (median of 8 months compared to 17 in the poorest quintile) [31].

Among the HIV-positive women in this study who chose to breastfeed, almost half of them stopped breastfeeding before 12 weeks. This is earlier than the national recommendation at the time of the study of abrupt cessation at four months [5]. Early cessation of breastfeeding amongst HIV infected infants has been shown to be detrimental to survival in a large randomised trial in Zambia [18]. Infected infants who were weaned from breast milk by five months had a 74% mortality rate at 24 months compared to 55% amongst those with prolonged breastfeeding [18]. This risk continued into the second year of life with weaning before 18 months of age associated with increased risk of child death compared to breastfeeding beyond 18 months [32]. An earlier study conducted at the same sites as the PROMISE study found that health workers suggesting formula use was the strongest predictor of complete breastfeeding cessation by 24 weeks amongst HIV-positive women [16]. Evidence from South Africa and Zambia led to a change in the WHO HIV and infant feeding recommendations in 2010 [33] which now state that at six months, if replacement feeding is still not acceptable, feasible, affordable, sustainable and safe, continuation of breastfeeding with additional complementary foods is recommended.

The lack of an effect of the peer counsellors on breastfeeding cessation raises questions regarding the quality of their counselling. Further qualitative research undertaken during the trial has highlighted that in some instances women feared the peer counsellor visits and questioned their intentions [34]. Under these circumstances it would be difficult for the peer counsellors to influence women’s feeding practices. In contrast, qualitative research undertaken in the Ugandan site of this trial, where the intervention had a large effect, found that over 95% of the women expressed satisfaction with the peer counselling they received [35]. Furthermore, in Uganda the peer counsellors were selected by their communities [36] while in South Africa a formal advertising, short listing and interview process was followed according to local employment processes [34].

In this study, almost a quarter of HIV-positive women and 10% of negative women experienced a breast health problem before six weeks postpartum. An intervention study in South Africa that provided regular home counselling to support exclusive breastfeeding found a low prevalence of breast health problems with no significant differences between HIV-positive and negative women[37]. As with our study, this study also found that women who had not exclusively breast-fed their infants were more likely to experience breast health problems compared with women who had exclusively breast-fed [37]. A similar finding has recently been reported from Zambia where non-exclusive breastfeeding resulted in twice the risk of experiencing a breast problem compared with exclusive breastfeeding [38]. Due to temporal uncertainty, reverse causality should be considered in understanding the relationship between breast health problems and breastfeeding cessation; did the infrequent/ceasing breastfeeding lead to breast problems or did the occurrence of breast problems lead to breastfeeding cessation?

Our study had several potential limitations. The sites where this research was undertaken were purposely selected and the infrastructural conditions differed greatly between them. They do, however, reflect a range in which many settings in South Africa could be comparable; either urban, peri-urban or rural areas. Pregnant women were recruited from within the communities with no other selection criteria except living in one of the study clusters, being pregnant and not planning to move away in the subsequent year. Postnatal inclusion criteria were a singleton live birth with no severe malformation which could interfere with breastfeeding. The HIV-positive sample in the study was fairly small, which limited our ability to conduct detailed sub group analysis. There may be other factors associated with breastfeeding cessation in our sample that we did not measure, such as advertising or promotion of formula in health facilities and communities. Further research, possibly qualitative in design, is needed to explore these aspects to understand more fully the drivers of breastfeeding cessation.

The study also has several strengths compared to many studies assessing infant feeding. The total study sample was large and infant feeding data were rigorously collected using both 24 hour and previous 7 day recall at four times during the first half of infancy.

In this study, despite a programme of home visitation in addition to routine facility infant feeding counselling, breastfeeding practices were poor. Improving breastfeeding practices in South Africa will require more effort including clear and consistent messages from health workers and community based workers and greatly improved breastfeeding support especially during the antenatal and early postnatal periods to ensure appropriate feeding choices, correct lactation techniques and optimal breastfeeding duration (see Table 3).

**Table 3:** Suggested actions for improving breastfeeding practices in South Africa

Community level actions	· Implement a national media and communication campaign for health workers and the general public promoting breastfeeding as a key intervention to reduce child mortality · Communicate the importance of early initiation of breastfeeding [39] · Communicate the benefits of exclusive breastfeeding for the first 6 months in HIV-positive and HIV-negative women [40][15]
Health service level actions	· Train all health workers in the benefits of exclusive breastfeeding, including doctors, nurses, dieticians and community health workers during initial training and reinforce during in-service training · Rapidly increase the proportion of hospitals with Baby Friendly status [41] · Employ breastfeeding counsellors in health facilities and at community level · Train existing community health workers to support breastfeeding and establish a system of home visits to women postpartum for lactation support · Avoid using formula milk in health facilities · No advertising of formula in health facilities
Policy level actions	· Restrict advertising/promotion of formula, including at professional conferences. · Review the current policy of provision of free formula milk to HIV-positive women [43] · Legally enforce the Code of Marketing of Breastmilk Substitutes [42]

## Conclusion

Increasing the duration of breastfeeding amongst women in South Africa is critical in order for the public health benefits of breastfeeding on child survival to be realised. There is an urgent need to develop and implement clear and consistent antenatal counselling messages to enable women to make appropriate infant feeding choices, especially for working women, together with early postnatal support to improve lactation techniques and prevent breast health problems.

## Competing interests

The authors declare they have no competing interests.

## Authors’ contributions

TD, DS, DJ, SS and CL planned and wrote the paper. SS and CL were study statisticians. DS, IE, EE and LF contributed to manuscript design and content. IE, WZ, MC, DJ and TD had particular responsibility for study implementation. TT was the central PI. All authors read and contributed towards the final draft.

## Pre-publication history

The pre-publication history for this paper can be accessed here:

http://www.biomedcentral.com/1471-2431/12/105/prepub
